# Educational Methods

**Published:** 1899-07

**Authors:** M. F. Ault

**Affiliations:** Dean of Central College of Dentistry, Indianapolis


### THE DENTAL REGISTER.

Vol. LIII.] JULY, 1899. [No. 7.

### COMMUNICATIONS.

### Educational Methods.

BY M. F. AULT, D.D.S., DEAN OF CENTRAL COLLEGE OF DENTISTRY, INDIANAPOLIS.

Read before the Indiana Dental Society.

*Mr. President and Members of the Indiana State Dental Association:*

Before attempting to practice dentistry the learner should make himself fully acquainted with the subject and endeavor to make his own the knowledge and skill of our best teachers and practitioners. An attempt to practice before there is acquaintance with the subject has as a consequence the discouragement, demoralization and failure which in no small degree characterize the profession. When there is discouragement, some part of the subject is not understood; when there is a feeling as if in the midst of demoralization, there is a lack of information by one or many in the profession, and when there is a failure it is due to individual ignorance or the influence of others who do not comprehend the entire subject. A full acquaintance with the subject demands from those in positions of power a careful study of two essential elements: *First, those who receive instruction, and secondly, those who impart instruction.*

Let us discuss briefly the first topic and make ourselves acquainted with some of the attributes which belong to an effective worker in school. The attribute of bodily comfort secures clearness of the thought, memory and will, and should be possessed by the person who is to receive instruction.

If a pupil can not be in a teachable mood after prolonged physical effort, or if the student can be directed on Monday so as to be unfit for duty on Tuesday, it is highly important that the Monday program should reduce the tension, under such work, to the lowest limit. A learner begins the work on Monday with an amount of energy which we will represent by $\frac{3}{3}$. The first hour is devoted to a lecture on anatomy, and it is safe to assume that one-third of the energy is expended in following the first lecture of the day. The second hour follows and attention is given to a lecture on operative dentistry; in this there is a dissipation of another third of energy. Query: Have we not reasoned ourselves into the belief that the hour from eleven to twelve is not a good one for a profound lecture? And yet, under the usual order this hour would be occupied in exhausting the remaining third of the student's energy by listening indifferently to such subjects as chemistry and physiology. Still, the afternoon work is to be met promptly, and the student feels that it should be satisfactory. During the noon hour, when food is taken, there is a very uncertain storage of energy for the afternoon duties. If the experiments made to determine the time of digestion are not at fault, the food taken at 12:30 could scarcely be appropriated before 3 o'clock, at which time we could physiologically expect a replenishing of vital force and on which the learner's physical ability is to depend for the next three hours. Now, after this decline, rise, and second decline of vital force, in what condition is the student to engage in mental effort, if he is ambitious enough to try it, or if the school management is illogical enough to expect it? Second query: Have we not reasoned ourselves into the belief that the vital force of the student does not warrant the usual night study if the mental action is to be clear and effective on Tuesday? We will say that the pupil feels that to keep up the work in a manner satisfactory to himself he must devote one-fourth of the sleeping time to study; and, judging from the way programs are arranged, the way lectures are poured out and the manual effort planned for the rest of the day, it is reasonable to suppose that for reviewing the points of the day the student would be forced to appropriate the time which nature intends for another purpose. So, as much mental work is to be conducted at the expense of meal time, the period of assimilation and sleeping time, there is nothing to be expected but to see the student appear on Tuesday morning in a disturbed condition and a decline in physical capacity for the duties of the day. And with an effort at repeating on Tuesday the work of Monday, he comes on Wednesday in a more agitated condition, and by the following Friday evening he is ready to abandon the effort of systematic review and the notes which are rapidly accumulating, and other assigned work must be postponed to some other time, he knows not when, but usually ends in the abnormal committing to memory which precedes examination.

If the argument presented thus far has any virtue, we are induced to believe that—

(*a*) The hour from 11 to 12 A.M is not a proper time for heavy lecture work, and when used should be for the least exacting work.

(*b*) In the event of afternoon lectures the student should not be specially taxed before 3 o'clock.

(*c*) The lectures should not be arranged so as to make after night work necessary.

(*d*) Instead of so many lectures as are usually provided, there should be regular study hours to take the place of night work, and the study hours should be devoted to lecture notes and assigned lessons in good text books.

(*e*) Instead of so many lecture hours there should be regular recitation hours presided over by a competent teacher and not left in the care of students.

I imagine I hear the objection that we do not have students as attentive and conscientious in the work as the picture drawn would indicate. I believe that pupils start in school with a desire to know the subject, that they experience a pleasure in their work, and that if their desire and pleasure is crushed and a surly, agitated feeling comes to pervade the college life, that it is due to an undue ambition and false theories in educational methods. The student is naturally all right, and whatever will conserve the enthusiasm of the early days and whatever will dispense with the high pressure which causes the flight of his laudable spirit should be allowed to prevail in the planning of a time bulletin. When I look at our usual college curriculum, and the way it is applied, there is presented to me the vision of a whirlwind. In this we see commotion, sand, leaves and dust under the control of distracted elements and no one able to tell their destiny, and in a very large degree it is the cyclonic character of school that brings the ashy hue to the face of a conscientious pupil. Listless recitation, bad digestion, ill feeling and revolt, the carouse and seeking lewd scenes, the indulgence in the lascivious, cheating in examination, parading as quacks and imposters, are all to be traced in a large degree to the high pressure and cyclonic nature of the modern curriculum.

A faculty consists of eight or ten individuals, and can it be rational for them to persist, hour after hour, day after day, and term after term, in no more than dumping great loads of facts and theories into the delicate receptacles of the mind, and failing to provide sufficient rest and periods of reflection? Haste and agitation destroys the most vital incentive of a student's career, and that is—*love of learning.* In order that the learner may acquaint himself with the subject, his education must be a healthy growth. A committing to memory, so as to show that the subject has not been considered in its many relations—just a reproduction of something stated and believed to be a good thing for examination—is no evidence of a healthy growth. Such knowledge is no more a part of the mind of the learner than a grain of wheat is a part of the cerebral tissue. There seems to be on the part of some who are styled scholars a determination not to pursue the subject for its own sake, but to pick out a few ideas which are believed to be useful, and in fact, only help the recipient to lead a superficial life. Our college course must be considered from the standpoint of human development and therefore be broad as it is, or else it must subscribe to a popular demand for the practical and therefore be narrowed to those few points which will enable the learner to acquire money in the least possible time.

Those who love learning will believe in the fullness of the dental course and think the time for its acquirement too short, while those who are devoid of the divine insight will do only that which is expedient. It is evident that there is something that must sway the student irrespective of his natural and acquired ability, and this something we call the *love of learning.* So to keep alive this vital spark is a very important question in arranging the college course, and the point of first consideration is how to provide for the bodily comfort by assigning the many duties at the proper time and to see that lectures are not too numerous and too long.

*The second attribute of the student is natural ability.* I have heard considerable comment about schools admitting many who are not naturally adapted to the work. Should applicants be required to show evidence of natural ability, judgment could be based upon several means of testing, but these would be of little practical value. Appearance could be made a test, the presentation of a drawing or some piece of work. Recommendation from one acquainted with the candidate's ingenuity, or by submitting to him questions which will draw out the natural instinct. As to the first test, we all know the deception of appearance. Mr. Garfield said that you do not know what possibilities may be buttoned up under a ragged coat. If the face should be made the criterion, no one is able to judge. To illustrate: I ask you to decide as to the eligibility of the two following persons, basing your decision upon facial expression: The first has low, sloping forehead, almost gorilla features, hair as close as a convict's, a pipe in his mouth, and looks in every way qualified for the penitentiary. The second example has a large, open countenance, requires a No. 7 hat, prominent frontal eminences, possesses the cranial form of Melancthon and seems well endowed to guide wayward men into the haven of rest. Will some one who is rather particular as to the natural adaptibility of students please tell which of the above is best fitted by nature for a dental student? We will imagine that the decision has been made. Now, I know these men personally and state the truth when I say the one who appears to be endowed for the State's prison is to-day engaged in masonry and is laying accurate brick and stone work, and the one possessing the face with strong marks is driving an express wagon. Another living example, in which I refrain from specific description, is a person who possessed in personal appearance but little to encourage him, and so far as his career during the freshman year was concerned there was not much to enable the school authorities to encourage his continuance. (I make this point in answer to the idea that if a pupil does not give evidence of success at the close of the first year he should be advised to withdraw.) My memory of this student is, at the close of the senior year, when I inspected his work upon a lower incisor, he restored nearly half the tooth with gold, his means of retention were good, the condensation was complete and the finish beautiful. Now, who can say that any school has the moral right to discourage such an aspirant?—a young man who in the beginning had no personal address to recommend him, and yet, so far as results were concerned in operating in one of the most difficult locations and a restoration of the most trying character, he closed his college career triumphantly.

*The third attribute necessary for the learner is acquired ability.* No phase of school work is receiving more attention than that of preliminary education, and so far has the question been urged that certain relations are much strained. There is no equitable policy in a plan which would prove disastrous to either the school or the student, and if the standard is placed too high it becomes prohibitory, providing there is an honest enforcement. A rule should not be adopted which can not be honestly applied—such a course brings all good rules into disregard. If students who possess good common educations and can verify it by good examinations are ruled out because the demands are above a *common English education*, then the school will be forced to raise its tuition proportionately. A large amount of money is needed to pay the incidental expenses of a college, to say nothing of a just compensation for those who devote sufficient time to make it a real educational place. So, should the restriction upon attendance be such as to reduce it one-half, it is evident the tuition would need to be doubled, and I have but little doubt that if the latest requirement is *honestly* enforced that the attendance will be reduced 66 percent, so that if the present tuition of $100 is necessary to keep a college in the best condition, those students who are able to pass the requirements would be called upon to pay $200 tuition. This must follow the law of self-preservation, and elimination must be followed by increase in tuition, otherwise the school must be run at a loss or cease to exist. Of course we may argue that the smaller the attendance the greater the advantage to those who attend; there will not be crowding, better opportunity for personal attention, and that a clinic attendance of 100 for twenty-five students is preferable to the same clinic for seventy-five students. This is no doubt true, but unless the students have strong financial support the payment of two or three times the present tuition would be a great hardship, if it would not preclude the attendance of those who pass the preliminary requirements. Suppose I should make the prophecy that every college without State support would fall under the honest application of the new preliminary rule; and yet this is what I see, unless the advance in tuition would obviate the difficulty, and this is certainly of very doubtful propriety. Suppose the rule is allowed to stand and the colleges publish it in their catalogues, and things move on and no crash comes—what has become of our prophecy? Simply this: that we have been cursed with the same old pretense of being good, and a searchlight upon entrance examinations would reveal the same old hypocrisy. Why can we not have a regulation that can be honestly enforced and bring about a belief that a person who can pass a good examination in the eight common English branches is a *good scholar*? There is sufficient vigor even in the common English requirement to rule out 25 percent of the applicants; but this I am willing to advocate and willing to apply, since the public school system provides for such training and a student who possesses it will be able to understand the presentation of the recognized dental course.

If we should assert that the time is not here for a high school requirement, what reasons could be offered to sustain the assertion?

(1st.) The plan cannot be honestly enforced.

(2nd.) High schools are not yet provided in all the townships.

(3rd.) There would be so few who graduate in high schools who would want to study dentistry.

(4th.) The ratio of males who do graduate is so low that there would not be at present enough students to justify the school in continuing.

And, as an additional reason, let us suppose a case which will probably be near the truth. There are ninety-two counties in Indiana. Suppose they will average one high school each and that for 1899 the classes averaged twenty graduates, this would furnish the schools 1,840 graduates to draw from; suppose that only 5 percent of the number are males and (a study of classes will show how few boys do graduate compared to girls) this percent equals ninety-two male graduates for the year. Suppose further that only 2 percent of the males take the dental course, and we have eighteen boys for the schools in Indiana. Providing the eighteen would all be loyal to the colleges already established in the State there would be no more than enough in their tuition to pay the rent if either school should succeed in getting all of them, and counting that some would go to Chicago, some to Cincinnati and others to Louisville. It is probable that those coming into the State from Ohio, Illinois and Kentucky would no more than equal the outgo.

I think I hear the remark that there are many more high schools than we have taken into account, that most counties have two or three high schools in the small towns. These schools are not high schools—they will with very few exceptions furnish instruction to a few students in physical geography and algebra, but do not afford a high school course. I am speaking of approved high schools, such as the literary department at Ann Arbor would recognize, and I doubt if the counties in the State would average one approved high school. I know from experience that a class of fifty will remain intact until they finish the A grammar grade, and during the four years of high school the ratio between male and female is so reduced that the graduating class will number four boys and sixteen girls. The unusually large class graduated at the Indianapolis High School in 1898 numbered one hundred and twelve, and of these there were only twenty boys, and it is my recollection that the class of 1899, though larger, presented the same proportion.

Those throughout the State who are suffering from the distractions of the dental-parlor man, the originator of painless dentistry and the five-dollar-plate-man may censure the colleges for admitting certain students, but the high school standard will never control or eliminate quackery—it cannot do it any more than high-grade education can make a disciple or an apostle. Really, quackery in all phases of life as well as in dentistry will never be extirpated until the minds of men respond actively to the doctrine of "Blessed are the pure in heart, for they shall see God." Even in the absence of a rule regulating the acquired ability of students, colleges would not admit ignorant students to any great extent because ignorance is an obstruction to the uniformity which worthy schools would have in their classes and ignorant students cannot be helpful in sustaining a college reputation, and for these reasons alone an educational institution will display its natural instinct of self-preservation by guarding against ignorant applicants. Now, in order to be clearly understood, I wish to say that I am for the common school standard and since any person who can pass the regular examination in the eight English branches can secure a license to teach, it seems to me that a similar test ought to be sufficient for admission to dental schools, and while I do not issue a challenge if anybody thinks the standard which I am advocating is not an intellectual test I would have him go before any county superintendent and measure himself by answering the questions prepared by the State Board of Education.

There is another phase of enforcing limited attendance by drastic measures, and that is the belittling influence of soliciting students. If in dental practice it is disgraceful to solicit business, and according to the code of ethics it is, it seems to me it is just as degrading for a dean, secretary or any part of a college management to solicit students. The effect of solicitation by colleges is to shake the gravity of the institution, place the school under undue obligation to the students and in a sense places the control of the school in the hands of those solicited, because I believe that no one can use persuasive measurements without feeling that it is a betrayal of weakness, especially when the persuasion is based upon odious comparisons, and I think the student himself will reason that if they are willing to solicit and make terms that it is a symptom of weakness, and therefore may be led to make demands which the management does not like to refuse, and yet the nature of most such cases forbids them being granted. Certainly an investigation of this point will convince the most skeptical that soliciting students has a tendency to lower the character of the school as much as soliciting lowers the tone of private practice, and this excessive preliminary requirement encourages or forces some if not all do.

There are several other important characteristics of the student to which attention could profitably be given, such as an elevated ideal, self-reliance, perseverence, concentration, enthusiasm, patience and obedience. Obedience to the rules and regulations of the school is of very great importance, and one rule which is much abused is that relating to work outside the school. Work at the student's room or in an office is a disturbing element. Experience teaches that when the mind of the learner is engaged on outside work that concentration upon the college work and obedience to college instruction is impossible. A student's place is in school—all his time being applied to the prescribed work, and commendable as is the effort of a few students to demonstrate native ability in the making of original appliances, it should not be encouraged at the expense of loyalty to the regular course. There is never time for foreign work and satisfactory college work at the same time, and these principles must be absolute and not be brought under control by a mercenary motive or a laudable aspiration. Education is often defined as self-control and this being true, no school can hope to educate and tolerate insubordination at the same time.

As I desire not to be too laborious, I will not explain my interpretation of the other features of the student's character but will outline some things which should receive more attention from those who impart instruction. A teacher should feel the inspiration which comes through a full acquaintance with the dentist's professional qualifications. These qualifications may be designated as follows:

1. A correct idea of his work.
2. Knowledge of the human, the constitution.
3. Acquaintance with educational means.
4. Tact in management of cases.
5. Insight into business.
6. Industry.
7. Manipulative skill.
8. Artistic taste.
9. Self-control.
10. Honesty.

Of course, we can only hint at these points and will pass them by saying that the learner cannot become acquainted with them unless the teacher is inspired from experience and bears the knowledge to him in the nature of glad tidings.

He should be taught that any dentist will feel crippled that cannot beget confidence in his ability. Let him be taught that a display of learning and assumptions of superiority are bad taste, and that a truly learned person is humble. Teach him that we live in stirring times, and it is necessary to see what is passing. Teach him that ill-chosen words and pointless remarks will never win the esteem of the patient, and that the greatest watchfulness is necessary to avoid narrowness and meanness. Let the teacher impress the learner with the proper incentives for his life task and for clearness. Let us enumerate what we deem worthy incentives:

(*a.*) To save the natural teeth.
(*b.*) To restore with artificial substitutes.
(*c.*) To gain knowledge from experience.
(*d.*) To discipline the hand.
(*e.*) To gratify aspiration.
(*f.*) To secure efficiency.
(*g.*) To excel competitors.

(*h.*) To co-operate with competitors.
(*i.*) Fear of the patient's report.
(*j.*) Freedom from shame.
(*k.*) Financial gain.
(*l.*) Approbation of the patient.
(*m.*) Approbation of the community.
(*n.*) Attainment of honor.
(*o.*) Duty of self-accomplishment.
(*p.*) Prospect of future reward.

I do not think it well to dwell upon the gloomy side of life. The teacher should be the cool and refreshing stream from which the pupil drinks the beautiful and abiding things of life. I think depression comes from teaching that:

(1.) Dentists are poor business men.
(2.) Expenses are out of proportion to income.
(3.) Practitioners usually begin with a debt.
(4.) Years come and go and find the dentist in debt.
(5.) The end comes and the family has only a legacy of debt.

If the first is true let the teacher explain how to be a good business man. If expenses are out of proportion to income teach the student all about expenses and show him what is a just income. If years come and go and the dentist is found in debt, the college should help to furnish the wisdom which cancels indebtedness and if it be true that the end comes and the family has only a legacy of debt, all the influences which work for the development of character are accountable. Oh, teacher, great is thy responsibility, but how the burdens in life can be neutralized by seeing the beauty in a full acquaintance with the subject, and knowing that you are the agent of the Divine will.

---

A REGULATION against spitting in public buildings, churches, theaters, markets, railway stations, and cars, has been made by the Boston Board of Health. It is hoped that the courts will not nullify this, as has been done elsewhere with a similar regulation in at least one of our large cities. The "spitting boy," as the public expectorator seems to be called in the Boston papers, certainly ought to be restricted.

